# (*Z*)-3-[2-(2,4-Di­nitro­phen­yl)hydrazin-1-yl­idene]isobenzo­furan-1(3*H*)-one di­chloro­methane hemisolvate

**DOI:** 10.1107/S1600536814004929

**Published:** 2014-03-12

**Authors:** Palak Agarwal, Pragati Mishra, Nikita Gupta, Neelam, Priyaranjan Sahoo, Satish Kumar

**Affiliations:** a Department of Chemistry, St. Stephen’s College, University Enclave, Delhi, 110007, India

## Abstract

In the title compound, 2C_14_H_8_N_4_O_6_·CH_2_Cl_2_, the di­chloro­methane solvent mol­ecule resides on a crystallographic twofold axis. The mean plane of the phthaliso­imide ring is oriented at a dihedral angle of 32.93 (12)° with respect to the nitro-substituted benzene ring. An intra­molecular N—H⋯O hydrogen bond occurs. The crystal packing features a short Cl⋯O halogen-bond inter­action [3.093 (3) Å].

## Related literature   

For a general background, see: Kaufmann (1927 ▶); Maekawa & Nanya (1959 ▶). For the preparation of hydrazone derivatives of phthalic anhydride, see: Chen *et al.* (1990 ▶). For halogen bond inter­actions, see: Gonnade *et al.* (2008 ▶); Metrangalo & Resnati (2007 ▶); Pedireddi *et al.* (1992 ▶). For a related structure, see: Guirado *et al.* (1997 ▶).
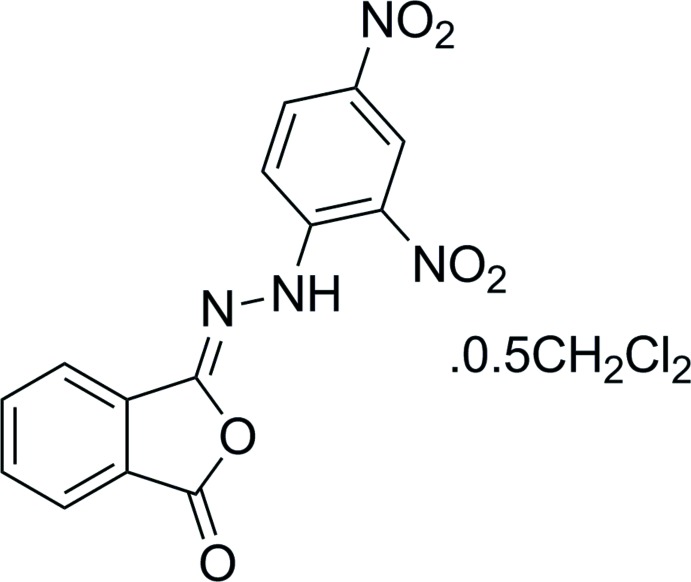


## Experimental   

### 

#### Crystal data  

2C_14_H_8_N_4_O_6_·CH_2_Cl_2_
*M* *_r_* = 741.41
Monoclinic, 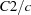

*a* = 14.0834 (11) Å
*b* = 8.2605 (6) Å
*c* = 26.561 (2) Å
β = 93.816 (7)°
*V* = 3083.2 (3) Å^3^
*Z* = 4
Mo *K*α radiation
μ = 0.29 mm^−1^
*T* = 297 K
0.40 × 0.40 × 0.15 mm

#### Data collection  

Agilent Xcalibur Sapphire3 diffractometer
Absorption correction: multi-scan (*CrysAlis PRO*; Agilent, 2011 ▶) *T*_min_ = 0.824, *T*_max_ = 1.000
20814 measured reflections
3832 independent reflections
2568 reflections with *I* > 2σ(*I*)
*R*_int_ = 0.039

#### Refinement  

*R*[*F* ^2^ > 2σ(*F* ^2^)] = 0.057
*wR*(*F* ^2^) = 0.141
*S* = 1.03
3832 reflections
234 parameters
Only H-atom displacement parameters refined
Δρ_max_ = 0.44 e Å^−3^
Δρ_min_ = −0.35 e Å^−3^

### 

Data collection: *CrysAlis PRO* (Agilent, 2011 ▶); cell refinement: *CrysAlis PRO*; data reduction: *CrysAlis PRO*; program(s) used to solve structure: *SHELXS97* (Sheldrick, 2008 ▶); program(s) used to refine structure: *SHELXL97* (Sheldrick, 2008 ▶); molecular graphics: *OLEX2* (Dolomanov *et al.*, 2009 ▶) and *Mercury* (Macrae *et al.*, 2008 ▶); software used to prepare material for publication: *OLEX2* and *publCIF* (Westrip, 2010 ▶).

## Supplementary Material

Crystal structure: contains datablock(s) I. DOI: 10.1107/S1600536814004929/fj2664sup1.cif

Structure factors: contains datablock(s) I. DOI: 10.1107/S1600536814004929/fj2664Isup2.hkl

Supporting information file. DOI: 10.1107/S1600536814004929/fj2664Isup3.cdx

Supporting information file. DOI: 10.1107/S1600536814004929/fj2664Isup4.cml

CCDC reference: 989735

Additional supporting information:  crystallographic information; 3D view; checkCIF report

## Figures and Tables

**Table 1:** Hydrogen-bond geometry (Å, °)

*D*—H⋯*A*	*D*—H	H⋯*A*	*D*⋯*A*	*D*—H⋯*A*
N6—H6⋯O3	0.81 (3)	1.99 (3)	2.613 (3)	134 (2)

**Fig. 1.**
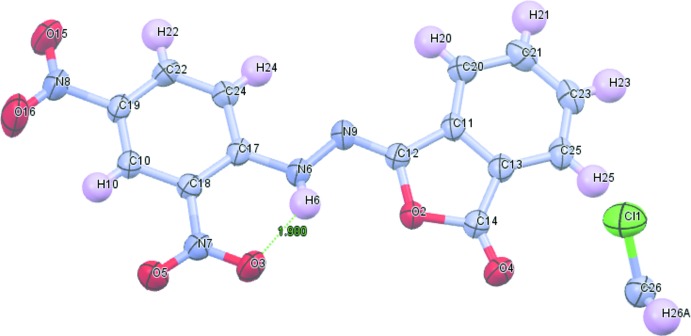
The molecular structure of the title compound, with atom labels and 50% probability displacement ellipsoids for non-H atoms.

**Fig. 2.**
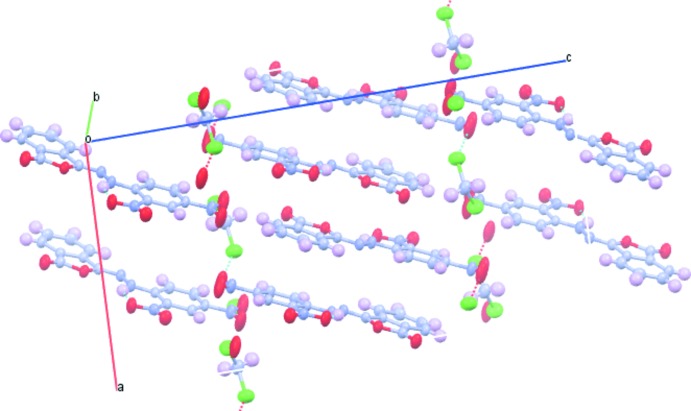
The packing diagram of the title compound viewed viewed along b axis, showing short intermolecular O···Cl halogen bonds and intramolecular N—H···O hydrogen bonds.

## supplementary crystallographic information

### 1. Comment

Heterocyclic compounds are useful for their ion binding, medicinal and insecticidal properties. The title compound was synthesized as a side product in an effort directed towards development of colorimetric anion sensors and its crystal structure is reported here. The reaction involved treatment of 2,4-dinitrophenylhydrazine with phthaloyl chloride in presence of triethylamine as a base. There are only a few reports on the preparation of hydrazone derivatives of phthalic anhydride in the literature (Chen *et al.*, 1990). The asymmetric unit (Fig. 1) consists of the title compound solvated with half a molecule of dichloromethane which lies on the crystallographic 2-fold axis. The phthalisoimides aromatic ring is nearly coplanar with nitroaromatic ring with a dihedral angle of 32.93 (C23—C11—C17—C10). A intramolecular hydrogen bond O3···H6 is also present in the title compound (Table 1). The crystal packing is stabilized by short Cl···.O halogen bond interaction (Fig 2) as reported in the literature (Gonnade *et al.*, 2008), (Pedireddi *et al.*, 1992), Metrangalo *et al.*, 2007).

### 2. Experimental

2,4-Dinitrophenylhydrazine (1.51 g, 7.65 mmol), dichloromethane (20 ml) and triethylamine (1 ml, 7.20 mmol) were taken in a 100 ml round bottom flask equipped with a magnetic stirrer bar. Phthalolyl chloride (0.5 ml, 2.46 mmol) was added to the stirred reaction mixture in a dropwise manner. The reaction mixture was stirred for 12 h and the precipitate obtained were filtered. The filtrate was added to 50 ml water. The organic layer was separated and washed thrice with 50 ml portion of 10% NaHCO_3_ solution followed by water. Organic layer was dried over sodium sulfate and kept overnight to yield light yellowish red crystals of the title compound as the side product of the reaction. Melting point 94–95°C.

### Crystal data

**Table:**

2C_14_H_8_N_4_O_6_·CH_2_Cl_2_	*F*(000) = 1512
*M**_r_* = 741.41	*D*_x_ = 1.597 Mg m^−^^3^
Monoclinic, *C*2/*c*	Mo *K*α radiation, λ = 0.71073 Å
*a* = 14.0834 (11) Å	Cell parameters from 3399 reflections
*b* = 8.2605 (6) Å	θ = 3.0–29.2°
*c* = 26.561 (2) Å	µ = 0.29 mm^−^^1^
β = 93.816 (7)°	*T* = 297 K
*V* = 3083.2 (3) Å^3^	Rect. prism, clear yellow–red
*Z* = 4	0.4 × 0.4 × 0.15 mm

### Data collection

**Table:**

Agilent Xcalibur Sapphire3 diffractometer	3832 independent reflections
Radiation source: Enhance (Mo) X-ray Source	2568 reflections with *I* > 2σ(*I*)
Graphite monochromator	*R*_int_ = 0.039
Detector resolution: 15.9853 pixels mm^-1^	θ_max_ = 29.3°, θ_min_ = 3.0°
ω scans	*h* = −19→19
Absorption correction: multi-scan (*CrysAlis PRO*; Agilent, 2011)	*k* = −11→10
*T*_min_ = 0.824, *T*_max_ = 1.000	*l* = −35→36
20814 measured reflections	

### Refinement

**Table:**

Refinement on *F*^2^	Primary atom site location: structure-invariant direct methods
Least-squares matrix: full	Hydrogen site location: mixed
*R*[*F*^2^ > 2σ(*F*^2^)] = 0.057	Only H-atom displacement parameters refined
*wR*(*F*^2^) = 0.141	*w* = 1/[σ^2^(*F*_o_^2^) + (0.053*P*)^2^ + 3.247*P*] where *P* = (*F*_o_^2^ + 2*F*_c_^2^)/3
*S* = 1.03	(Δ/σ)_max_ < 0.001
3832 reflections	Δρ_max_ = 0.44 e Å^−^^3^
234 parameters	Δρ_min_ = −0.35 e Å^−^^3^
0 restraints	

### Special details

**Table:**

Geometry. All e.s.d.'s (except the e.s.d. in the dihedral angle between two l.s. planes) are estimated using the full covariance matrix. The cell e.s.d.'s are taken into account individually in the estimation of e.s.d.'s in distances, angles and torsion angles; correlations between e.s.d.'s in cell parameters are only used when they are defined by crystal symmetry. An approximate (isotropic) treatment of cell e.s.d.'s is used for estimating e.s.d.'s involving l.s. planes.
Refinement. Refinement of *F*^2^ against ALL reflections. The weighted *R*-factor *wR* and goodness of fit *S* are based on *F*^2^, conventional *R*-factors *R* are based on *F*, with *F* set to zero for negative *F*^2^. The threshold expression of *F*^2^ > σ(*F*^2^) is used only for calculating *R*-factors(gt) *etc*. and is not relevant to the choice of reflections for refinement. *R*-factors based on *F*^2^ are statistically about twice as large as those based on *F*, and *R*- factors based on ALL data will be even larger.

### Fractional atomic coordinates and isotropic or equivalent isotropic displacement parameters (Å^2^)

**Table:**

	*x*	*y*	*z*	*U*_iso_*/*U*_eq_	
Cl1	0.39898 (6)	0.54376 (10)	0.73756 (3)	0.0797 (3)	
O2	0.41801 (11)	0.47047 (18)	0.54833 (5)	0.0411 (4)	
O3	0.30079 (13)	0.1587 (2)	0.45274 (6)	0.0566 (5)	
O4	0.48996 (13)	0.4056 (2)	0.62423 (6)	0.0562 (5)	
O5	0.24791 (13)	0.0449 (2)	0.38349 (7)	0.0554 (5)	
N6	0.33004 (14)	0.4709 (2)	0.45706 (7)	0.0407 (4)	
H6	0.3319 (18)	0.386 (3)	0.4723 (9)	0.049*	
N7	0.26939 (13)	0.1660 (2)	0.40803 (7)	0.0408 (4)	
N8	0.16035 (16)	0.4760 (3)	0.26002 (7)	0.0529 (5)	
N9	0.35397 (13)	0.6171 (2)	0.47867 (6)	0.0403 (4)	
C10	0.21556 (15)	0.3263 (3)	0.33550 (8)	0.0381 (5)	
H10	0.1961	0.2307	0.3195	0.046*	
C11	0.42937 (14)	0.7496 (3)	0.55314 (8)	0.0357 (5)	
C12	0.39564 (15)	0.6131 (3)	0.52246 (8)	0.0371 (5)	
C13	0.47111 (15)	0.6872 (3)	0.59783 (8)	0.0377 (5)	
C14	0.46430 (15)	0.5103 (3)	0.59583 (8)	0.0400 (5)	
O15	0.1569 (2)	0.6044 (3)	0.23776 (8)	0.0904 (8)	
O16	0.13589 (19)	0.3503 (3)	0.23967 (7)	0.0891 (8)	
C17	0.28732 (14)	0.4683 (3)	0.40931 (7)	0.0355 (5)	
C18	0.25776 (14)	0.3240 (3)	0.38441 (8)	0.0351 (5)	
C19	0.20337 (15)	0.4720 (3)	0.31152 (8)	0.0396 (5)	
C20	0.42629 (17)	0.9146 (3)	0.54405 (9)	0.0443 (5)	
H20	0.3976	0.9565	0.5143	0.053*	
C21	0.46757 (18)	1.0142 (3)	0.58095 (10)	0.0508 (6)	
H21	0.4676	1.1255	0.5757	0.061*	
C22	0.23198 (16)	0.6165 (3)	0.33451 (8)	0.0440 (5)	
H22	0.2230	0.7141	0.3174	0.053*	
C23	0.50939 (17)	0.9519 (3)	0.62607 (9)	0.0495 (6)	
H23	0.5361	1.0227	0.6503	0.059*	
C24	0.27340 (16)	0.6143 (3)	0.38250 (8)	0.0410 (5)	
H24	0.2928	0.7112	0.3978	0.049*	
C25	0.51179 (16)	0.7874 (3)	0.63543 (9)	0.0449 (6)	
H25	0.5393	0.7456	0.6655	0.054*	
C26	0.5000	0.4238 (5)	0.7500	0.0587 (10)	
H26	0.4913	0.3631	0.7795	0.070*	

### Atomic displacement parameters (Å^2^)

**Table:**

	*U*^11^	*U*^22^	*U*^33^	*U*^12^	*U*^13^	*U*^23^
Cl1	0.0687 (5)	0.0757 (5)	0.0948 (6)	0.0155 (4)	0.0049 (4)	0.0027 (4)
O2	0.0502 (9)	0.0379 (8)	0.0340 (8)	0.0005 (7)	−0.0059 (7)	−0.0030 (7)
O3	0.0822 (13)	0.0478 (10)	0.0378 (9)	0.0001 (9)	−0.0120 (8)	0.0103 (8)
O4	0.0750 (12)	0.0465 (10)	0.0451 (10)	0.0060 (9)	−0.0122 (8)	0.0055 (8)
O5	0.0755 (12)	0.0371 (9)	0.0523 (10)	−0.0031 (8)	−0.0053 (9)	−0.0018 (8)
N6	0.0515 (11)	0.0388 (11)	0.0306 (10)	−0.0002 (9)	−0.0056 (8)	−0.0004 (8)
N7	0.0447 (10)	0.0401 (11)	0.0371 (10)	0.0008 (8)	−0.0003 (8)	0.0028 (8)
N8	0.0681 (14)	0.0512 (13)	0.0371 (11)	0.0025 (11)	−0.0126 (10)	0.0015 (10)
N9	0.0465 (10)	0.0418 (11)	0.0321 (9)	0.0003 (8)	−0.0013 (8)	−0.0045 (8)
C10	0.0406 (11)	0.0419 (12)	0.0311 (11)	0.0021 (10)	−0.0026 (9)	−0.0042 (9)
C11	0.0364 (10)	0.0406 (12)	0.0300 (10)	0.0003 (9)	0.0015 (8)	−0.0046 (9)
C12	0.0407 (11)	0.0391 (12)	0.0312 (11)	0.0045 (9)	−0.0001 (9)	−0.0010 (9)
C13	0.0372 (11)	0.0420 (12)	0.0339 (11)	0.0013 (9)	0.0011 (9)	−0.0034 (9)
C14	0.0418 (12)	0.0427 (13)	0.0348 (11)	0.0023 (10)	−0.0019 (9)	−0.0021 (10)
O15	0.151 (2)	0.0621 (13)	0.0527 (12)	0.0099 (14)	−0.0355 (13)	0.0106 (10)
O16	0.142 (2)	0.0669 (14)	0.0526 (12)	−0.0249 (14)	−0.0391 (13)	0.0023 (11)
C17	0.0340 (10)	0.0435 (12)	0.0287 (10)	0.0018 (9)	−0.0002 (8)	−0.0007 (9)
C18	0.0366 (10)	0.0362 (11)	0.0325 (11)	0.0033 (9)	0.0021 (8)	0.0006 (9)
C19	0.0421 (12)	0.0476 (13)	0.0282 (11)	0.0032 (10)	−0.0045 (9)	−0.0002 (10)
C20	0.0512 (13)	0.0395 (13)	0.0422 (12)	0.0028 (10)	0.0029 (10)	0.0016 (10)
C21	0.0580 (15)	0.0380 (13)	0.0570 (15)	−0.0044 (11)	0.0085 (12)	−0.0060 (11)
C22	0.0537 (14)	0.0413 (13)	0.0360 (12)	0.0056 (11)	−0.0032 (10)	0.0068 (10)
C23	0.0505 (14)	0.0505 (15)	0.0475 (14)	−0.0073 (11)	0.0028 (11)	−0.0169 (12)
C24	0.0489 (13)	0.0360 (12)	0.0374 (12)	0.0006 (10)	−0.0028 (10)	−0.0018 (9)
C25	0.0441 (12)	0.0541 (15)	0.0356 (12)	−0.0018 (11)	−0.0033 (9)	−0.0085 (10)
C26	0.070 (2)	0.052 (2)	0.053 (2)	0.000	−0.0091 (18)	0.000

### Geometric parameters (Å, º)

**Table:**

Cl1—C26	1.747 (2)	C11—C20	1.385 (3)
O2—C12	1.389 (3)	C13—C14	1.466 (3)
O2—C14	1.419 (3)	C13—C25	1.391 (3)
O3—N7	1.241 (2)	C17—C18	1.413 (3)
O4—C14	1.188 (3)	C17—C24	1.407 (3)
O5—N7	1.221 (2)	C19—C22	1.389 (3)
N6—H6	0.81 (3)	C20—H20	0.9300
N6—N9	1.370 (3)	C20—C21	1.378 (3)
N6—C17	1.367 (3)	C21—H21	0.9300
N7—C18	1.453 (3)	C21—C23	1.398 (4)
N8—O15	1.214 (3)	C22—H22	0.9300
N8—O16	1.210 (3)	C22—C24	1.366 (3)
N8—C19	1.459 (3)	C23—H23	0.9300
N9—C12	1.268 (3)	C23—C25	1.382 (3)
C10—H10	0.9300	C24—H24	0.9300
C10—C18	1.392 (3)	C25—H25	0.9300
C10—C19	1.367 (3)	C26—Cl1^i^	1.747 (2)
C11—C12	1.453 (3)	C26—H26	0.9440
C11—C13	1.388 (3)		
			
C12—O2—C14	108.59 (16)	C24—C17—C18	117.35 (18)
N9—N6—H6	123.9 (18)	C10—C18—N7	116.37 (19)
C17—N6—H6	116.5 (18)	C10—C18—C17	121.29 (19)
C17—N6—N9	118.88 (18)	C17—C18—N7	122.34 (18)
O3—N7—C18	118.66 (18)	C10—C19—N8	119.2 (2)
O5—N7—O3	122.09 (19)	C10—C19—C22	121.89 (19)
O5—N7—C18	119.25 (17)	C22—C19—N8	118.9 (2)
O15—N8—C19	118.5 (2)	C11—C20—H20	121.4
O16—N8—O15	122.1 (2)	C21—C20—C11	117.2 (2)
O16—N8—C19	119.2 (2)	C21—C20—H20	121.4
C12—N9—N6	116.54 (18)	C20—C21—H21	119.2
C18—C10—H10	120.7	C20—C21—C23	121.6 (2)
C19—C10—H10	120.7	C23—C21—H21	119.2
C19—C10—C18	118.7 (2)	C19—C22—H22	120.3
C13—C11—C12	107.21 (19)	C24—C22—C19	119.5 (2)
C20—C11—C12	131.4 (2)	C24—C22—H22	120.3
C20—C11—C13	121.4 (2)	C21—C23—H23	119.3
O2—C12—C11	109.01 (17)	C25—C23—C21	121.4 (2)
N9—C12—O2	123.53 (19)	C25—C23—H23	119.3
N9—C12—C11	127.5 (2)	C17—C24—H24	119.3
C11—C13—C14	108.41 (19)	C22—C24—C17	121.3 (2)
C11—C13—C25	121.6 (2)	C22—C24—H24	119.3
C25—C13—C14	130.0 (2)	C13—C25—H25	121.6
O2—C14—C13	106.77 (18)	C23—C25—C13	116.9 (2)
O4—C14—O2	119.9 (2)	C23—C25—H25	121.6
O4—C14—C13	133.3 (2)	Cl1—C26—Cl1^i^	110.9 (2)
N6—C17—C18	123.03 (19)	Cl1—C26—H26	108.0
N6—C17—C24	119.6 (2)	Cl1^i^—C26—H26	107.0
			
O3—N7—C18—C10	−175.26 (19)	C14—O2—C12—N9	179.8 (2)
O3—N7—C18—C17	4.7 (3)	C14—O2—C12—C11	1.0 (2)
O5—N7—C18—C10	4.6 (3)	C14—C13—C25—C23	178.5 (2)
O5—N7—C18—C17	−175.45 (19)	O15—N8—C19—C10	−174.2 (2)
N6—N9—C12—O2	0.6 (3)	O15—N8—C19—C22	5.0 (4)
N6—N9—C12—C11	179.12 (19)	O16—N8—C19—C10	0.4 (4)
N6—C17—C18—N7	0.8 (3)	O16—N8—C19—C22	179.5 (2)
N6—C17—C18—C10	−179.2 (2)	C17—N6—N9—C12	−178.2 (2)
N6—C17—C24—C22	179.2 (2)	C18—C10—C19—N8	179.22 (19)
N8—C19—C22—C24	−179.2 (2)	C18—C10—C19—C22	0.1 (3)
N9—N6—C17—C18	−178.27 (18)	C18—C17—C24—C22	0.7 (3)
N9—N6—C17—C24	3.3 (3)	C19—C10—C18—N7	−179.73 (18)
C10—C19—C22—C24	−0.1 (3)	C19—C10—C18—C17	0.3 (3)
C11—C13—C14—O2	0.5 (2)	C19—C22—C24—C17	−0.3 (3)
C11—C13—C14—O4	179.2 (3)	C20—C11—C12—O2	178.5 (2)
C11—C13—C25—C23	−0.5 (3)	C20—C11—C12—N9	−0.1 (4)
C11—C20—C21—C23	−1.1 (3)	C20—C11—C13—C14	−179.2 (2)
C12—O2—C14—O4	−179.9 (2)	C20—C11—C13—C25	−0.1 (3)
C12—O2—C14—C13	−1.0 (2)	C20—C21—C23—C25	0.5 (4)
C12—C11—C13—C14	0.1 (2)	C21—C23—C25—C13	0.3 (3)
C12—C11—C13—C25	179.23 (19)	C24—C17—C18—N7	179.32 (19)
C12—C11—C20—C21	−178.3 (2)	C24—C17—C18—C10	−0.7 (3)
C13—C11—C12—O2	−0.7 (2)	C25—C13—C14—O2	−178.5 (2)
C13—C11—C12—N9	−179.4 (2)	C25—C13—C14—O4	0.2 (4)
C13—C11—C20—C21	0.9 (3)		

Symmetry code: (i) −*x*+1, *y*, −*z*+3/2.

### Hydrogen-bond geometry (Å, º)

**Table:**

*D*—H···*A*	*D*—H	H···*A*	*D*···*A*	*D*—H···*A*
N6—H6···O3	0.81 (3)	1.99 (3)	2.613 (3)	134 (2)
